# Production of functional double‐stranded RNA using a prokaryotic expression system in *Escherichia coli*

**DOI:** 10.1002/mbo3.787

**Published:** 2018-12-27

**Authors:** Zhengjun Chen, Jindian He, Pan Luo, Xiangkai Li, Yuan Gao

**Affiliations:** ^1^ College of Life Science and Technology Gansu Agricultural University Lanzhou China; ^2^ MOE Key Laboratory of Cell Activities and Stress Adaptations, College of Life Sciences Lanzhou University Lanzhou China

**Keywords:** dsRNA, hpRNA, immersion of roots, LDH, RNAi

## Abstract

RNA interference (RNAi) is a nucleic acid metabolism system utilized for the post‐translational regulation of endogenous genes or for defense against exogenous RNA or transposable elements. Double‐stranded RNA (dsRNA)‐mediated RNAi shows broad application prospects to improve existing plant traits and combat invading pathogens or pests. To improve dsRNA transcriptional efficiency using a prokaryotic expression system, *Trxz* gene, an essential gene for the early development of chloroplasts in *Arabidopsis thaliana*, was chosen for a functional study. Two types of recombinant expression vectors, pDP‐*Trxz* and phP‐*Trxz*‐N/L, were constructed to generate dsTrxz, the dsRNA which specifically induces *Trxz* gene silencing. Gel electrophoresis tests showed that phP vectors performed better and produced more dsRNA than the pDP vector under the same conditions. Purification of dsTrxz by enzymatic digestion indicated that highly purified dsRNA can be obtained through the use of DNase enzymatic hydrolysis assay. To confirm the knockdown effect of the dsRNA, a root immersion assay was performed, and we found that the root immersion culture could continue to affect the growth and development of *A. thaliana*. This included inhibiting the development of new leaves, causing weak plant development, leaf whitening, and other symptoms. This indicated that in vitro expressed dsRNA can be absorbed through *Arabidopsis* roots and can continue to trigger *Trxz* gene silencing. To delay dsRNA degradation and extend the effectiveness of RNAi, nanomaterial layered double hydroxide (LDH)‐mediated BioClay was performed. We found that LDH‐mediated BioClay alleviates the degree of dsRNA degradation, which provides a new idea for the storage and transportation of dsRNA.

## 1 INTRODUCTION

RNA interference (RNAi) was originally found in plants and was considered as a natural antiviral defense system for them. Later, RNAi was found to be widely present in animals and microorganisms such as bacteria and fungi (Agrawal et al., 2003; Meister & Tuschl, 2004). Nowadays, RNAi is becoming a powerful approach that effectively suppresses or silences the expression of target genes (Dutta, Banakar, & Rao, 2014). In plants, fungi, and most invertebrates, RNAi is mediated by sequence‐specific double‐stranded RNA (dsRNA) (Al‐Ayedh, Rizwan‐Ul‐haq, Hussain, & Aljabr, 2016; Fellmann & Lowe, 2014). Therefore, full‐length dsRNA molecules are often used to regulate gene expression and to study functional genomics.

RNAi is triggered by exogenous or endogenous dsRNA. When long exogenous dsRNA fragments enter animal cells, they are first identified by the specificity of the sequence‐free selection of enzyme Dicer, a dsRNA specific endonuclease of the RNase III family. Dicer is a cohesive nucleotide that contains a helicase domain and an internal dimeric RNase III domain. This component differs among organisms. The enzyme that plays this role in plants is called dicer‐like (DCL) (Fukudome & Fukuhara, 2017). In host cells, long exogenous dsRNA is processed into small RNA fragments, termed as small interfering RNA (siRNA) by Dicer or DCL. siRNA, characterized by two unpaired nucleotide overhangs at the 5′‐ and 3′‐terminals (Zhao, Zhao, & Xia, 2005), binds to the RNA‐induced silencing complex (RISC) to function in the cells (Filipowicz, 2005; Tang, 2005).

RISC is composed of slicer and Argonaute proteins with RNase H‐domains. Argonaute proteins first mediate the unwinding of the siRNA and select one strand as the guide RNA (gRNA), which is key to subsequent gene silencing depending on siRNA secondary structures and its thermodynamic stability (Khvorova, Reynolds, & Jayasena, 2003). After the degradation of the nonselection strand, that is passenger RNA, by Argonaute, the RISC complex binds to mRNA under the guidance of gRNA in a sequence‐matched manner. It then initiates slicer‐mediated degradation of target mRNA, a RISC component (Zhao et al., 2005), resulting in post‐transcriptional gene silencing (PTGS) of target genes (Filipowicz, Jaskiewicz, Kolb, & Pillai, 2005; Moazed, 2009).

Notably, long hairpin RNA (hpRNA) is also a kind of dsRNA. Compared with perfectly matched dsRNA, hpRNA has a terminal stem‐loop, similar to pre‐miRNA (Arora, Rana, Chhabra, Jaiswal, & Rani, 2013). The terminal stem‐loop of hpRNA is cut by spliceosomes after entering the cells and forms a complete dsRNA to perform PTGS as dsRNA, although the mechanism of terminal stem‐loop cutting remains unclear (Okamura et al., 2008; Xiang, Fruehauf, & Li, 2006).

PCR, in vitro transcription based on double‐strand annealing, is a typical method to synthesize dsRNA molecules (Chalupnikova, Nejepinska, & Svoboda, 2013), although false amplification leads to poor quality dsRNA products. Transcription based on a prokaryotic expression system is another way to obtain high‐quality dsRNA. In this study, the M‐JM109‐LacY strain, an RNase III‐deficient *Escherichia coli*, was selected for dsRNA expression. This is because RNase III, transcribed from the Rnc gene in *E. coli*, is widely present and could block the production of dsRNA for its cleavage of long double‐stranded product, into short segments with the length of 12–15 bp (Court et al., 2013; Yin et al., 2009). The M‐JM109‐LacY strain is a mutant strain of JM109 (DE3) with knockout of Rnc and LacY genes, so it is deficient in RNase III activity and LacY permease, which is helpful to improve the efficiency of IPTG induction (Guan & Kaback, 2006).

## 2 METHOD AND MATERIALS

### 2.1 Target gene selection

In this study, the full‐length cDNA of *Arabidopsis thaliana Trxz* gene was selected as a target gene to verify the silencing effect of RNAi. The *TrxZ* gene, a plastidial thioredoxin (*Trxz*) between the x and y type TRXs, functions in the thio‐modification in chloroplasts (Collin et al., 2003). Recently, several independent studies showed that *Trxz* constitutes a subunit of plastid‐encoded RNA polymerase (PEP), and sufficient PEP production is essential for the early development of chloroplasts (Arsova et al., 2010; Bohrer et al., 2012). Inhibition of PEP expression will impact the early development of chloroplasts in the plant irreversibly. The *Trxz* mutant strain is a fully albino phenotype in *A. thaliana*; chloroplast development is suppressed, the leaves become yellowish white, and the plants develop weakly or even die later (Arsova et al., 2010; Yamazaki, Motohashi, Kasama, Hara, & Hisabori, 2004).

### 2.2 Construction of pDP‐*Trxz* recombinant vectors

In this study, pUC19 cloning vector was used as the backbone to construct recombinant vectors. PCR‐mediated point mutation was performed to remove the original PstI, EcoRI, and XbaI restriction sites on the pUC19 backbone. Meanwhile, bidirectional sequences of T7 promoter and two terminators were introduced into the plasmid backbone via PCR. The new vector was named pDP‐T. Then, the full‐length coding sequence of *Trxz* gene was cloned and directly inserted into pDP‐T to generate the pDP‐*Trxz* recombinant plasmid. The transcriptional product of pDP‐*Trxz* was termed as dsTrxz.

### 2.3 Construction of phP recombinant vector

For the construction of the phP recombinant vectors, including phP‐*Trxz*‐L and phP‐*Trxz*‐N vectors, pUC19 was used as the backbone. A tandem sequence including the T7 promoter, the *Trxz* sense, loop, *Trxz* antisense, and two terminator sequences was synthesized and inserted into the backbone plasmid using *Eco*RI and *Pst*I double‐enzyme digestion. The transcripts of phP recombinant vectors were termed as hpTrxz‐L and hpTrxz‐N, respectively.

### 2.4 Transcription and extraction of dsRNA

In this study, the M‐JM109 LacY strain was used for dsRNA synthesis. The optimized conditions with IPTG concentration (0.5–1 mM), induction temperature (37 ± 4°C), induction period, and LB media (1% tryptone, 0.5% yeast extract, 1% NaCl, pH: 7.2–7.4) were used in this study (Aalto et al., 2007; Goldoni, Azzalin, Macino, & Cogoni, 2004). The experimental procedures are described as follows. Recombinant plasmids were transformed into M‐JM109 LacY‐competent cells. dsRNA was induced in LB medium with 0.5 mM IPTG in a 37°C shaker at 180 rpm, and the bacteria solution was incubated until the value of OD_600_ was between 0.4 and 0.5.

An appropriate amount of induced bacteria was collected from centrifuging under 10,000 *g* for 1 min. The total RNA was extracted using phenol‐chloroform extraction (pH 8.0) (Tenllado, Martinez‐Garcia, Vargas, & Diaz‐Ruiz, 2003). When isopropanol and sodium acetate solution are finally added to precipitate RNA, an ice bath for at least 30 min is needed. Qiagen RDD buffer with DNase (GmBH, Germany) was used in this experiment as a solvent to prepare for subsequent enzymatic digestion. RNA concentration was analyzed with an Epoch Multi‐Volume Spectrophotometer system (BioTek, Winooski, VT, USA).

### 2.5 Purification of dsRNA by enzymatic digestion

In this experiment, crude extracted dsRNA was purified by enzymatic digestion, and dsRNA extracts were treated with DNase to hydrolyze and remove contamination of genomic DNA and plasmid DNA. It should be noted that all work starting from the extraction of dsRNA should be performed in a sterile, RNase (dicer)‐free environment. Sterile, RNase‐free solutions and materials should be used to protect the RNA products from degradation by external RNases.

### 2.6 Detection of dsRNA interference efficiency

To verify the interference efficiency of purified dsTrxz, the roots of *A. thaliana* seedlings were immersed with dsTrxz, as described in previous studies (Hunter, Glick, Paldi, & Bextine, 2012), and the RNAi efficiency of different dsTrxz was determined through the yellow flowering rate of the seedlings.

### 2.7 BioClay assay

**Figure A1:**
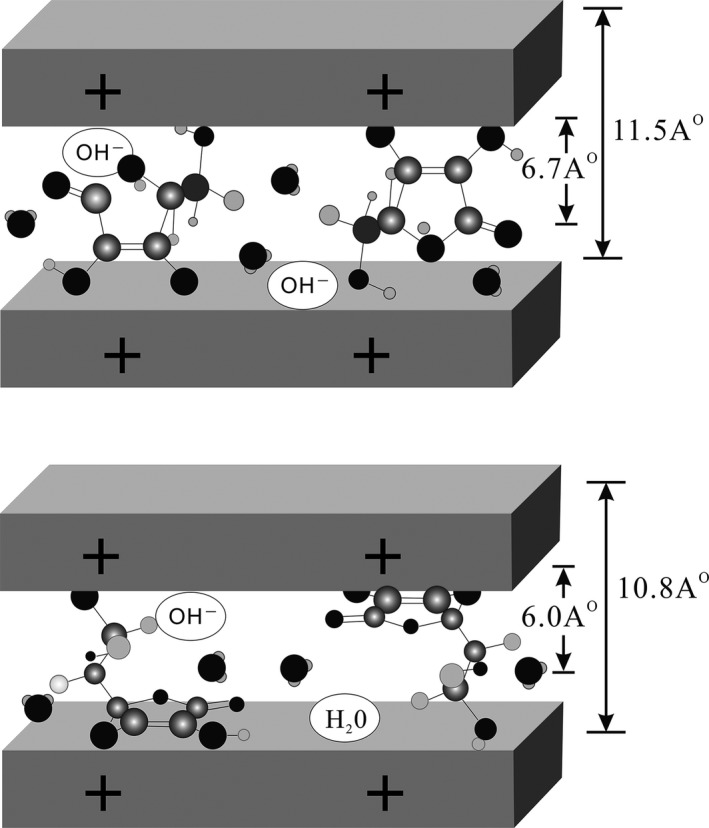
Molecular structure of layered double hydroxide

The exposed dsRNA molecules are highly susceptible to degradation by the environmental RNase under natural conditions. The emergence of nanomaterial layered double hydroxide (LDH) provides an efficient way to extend the life of dsRNA in the natural environment. The LDH nanosheets are inorganic layered material with a negative charge (see Appendix Figure A1). Its general expression is as follows: [(M^2+^(1 − *x*)M^3+^(OH^−^)_2_)*x* + (Am − *x*/*m* × nH_2_O)*^x^* ^−^] (Gasser, 2009). The properties of LDH determine that it is capable to store negatively charged molecules, such as dsRNA molecules with negatively charged phosphate groups.

BioClay is the formation of complex through the combination of LDH and dsRNA. In this study, purified dsRNA was dissolved into LDH solution (with a particle size of 172 nm) and double distilled water (ddH_2_O), respectively, to a final concentration of 20 ng/μl. All samples were kept at room temperature and collected at days 0, 5, 10, 17, and 21 for agarose gel electrophoresis. Quantitative analysis of gel bands was performed by ImageJ (NIH‐NCBI, Bethesda, MD, USA) as previous described (Gao et al., 2015).

### 2.8 Statistical analysis

Numerical data are presented as mean ± standard deviation (*SD*) and were analyzed with one‐way ANOVA. *p*‐values of <0.05 were considered to be significant.

## 3 RESULTS

### 3.1 Vector design and construction

**Figure 1:**
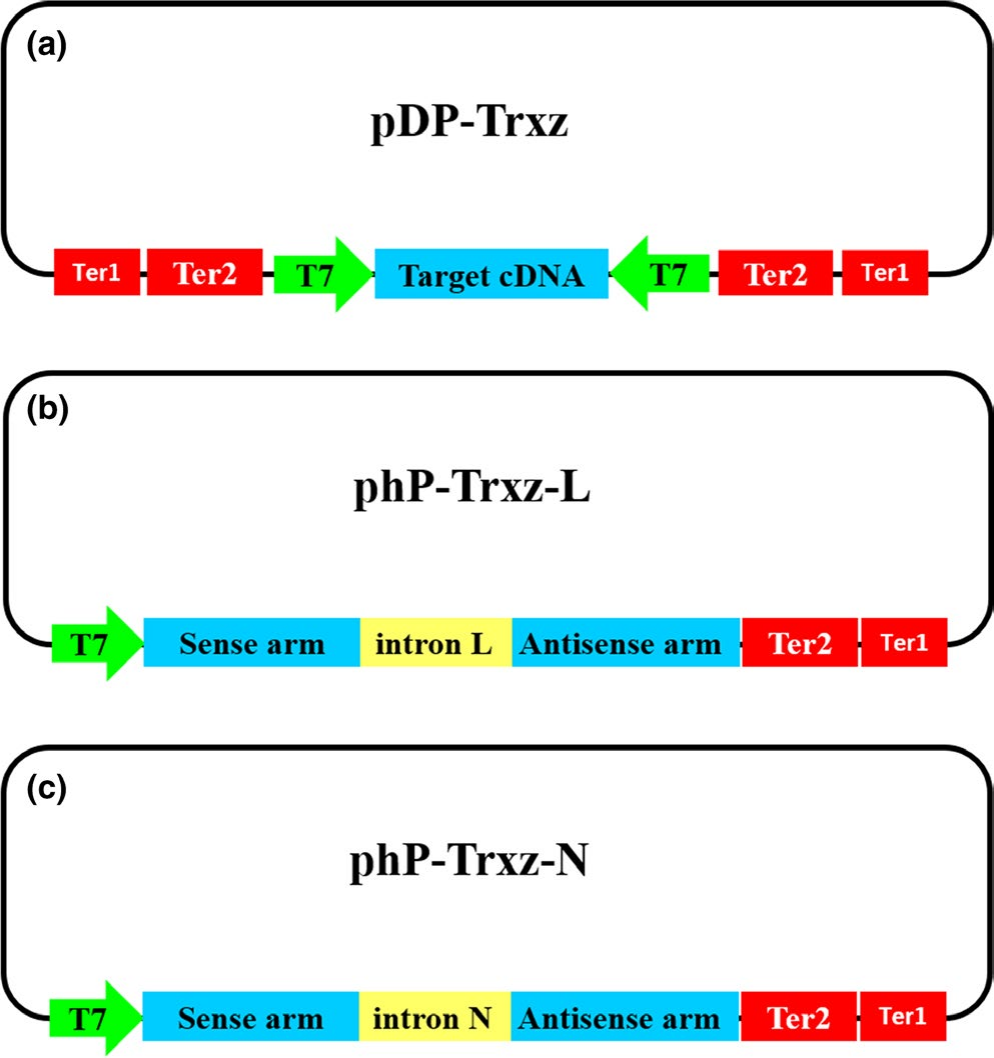
Double promoter recombination vector pDP‐*Trxz* (a) and hairpin transcriptional vector phP‐*Trxz*‐L (b) and phP‐Trxz‐N (c) construct. The arrows indicate the direction of transcription. All plasmids were based on pUC19 cloning vectors. T7: T7 promoter. Ter, terminator

To achieve efficient expression of dsRNA, a specific dsRNA expression vector, pDP, containing two T7 promoters, was generated (Figure 1a). These two T7 promoters are located opposite one another on both sides of the multiple cloning site, and two terminators were added on the outside of the two T7 promoters, respectively.

In contrast, the hpRNA expression vector, phP, contains a T7 promoter, the sense and antisense strands of the target sequence, and a loop (Figure 1b,c). The antisense sequence is used to form a terminal stem‐loop structure. In this study, two hpRNA expression vectors were designed, with the only difference being the loop sequence connecting the sense and antisense strands. In phP‐L vector, the loop is the noncoding region of the chloramphenicol‐resistance gene with a length of 120 bp: 5′‐ATTTTAGATTCCAACCTATGGAACTGATG AATGGGAGCAGTGGTGGAATGCCTTTAATGAGGAAAACCTGTTTTGCTCAGAAGAAATGCCATCTAGTGATGATGAGGCTACTGCTGACTC‐3′. dsRNA produced by phP‐L vector was named hpTrxz‐L (Figure 1b). In phP‐N vector, an intron of the *A. thaliana* FAD2 gene was selected as the loop sequence, 106 bp in length: 5′‐CCGAAACAAAGTAATTACAGTAACTTGCACTTCAAGGAATCTTGAGTTTCGCTTTTTATTTGTAAGTTTTGTAGCCACTGATATAATTTTTTGCAGGTACATAGAG‐3′. dsRNA produced by phP‐N vector was named hpTrxz‐N (Figure 1c).

### 3.2 Detection of dsRNA expression efficiency in pDP and phP vectors

**Figure 2:**
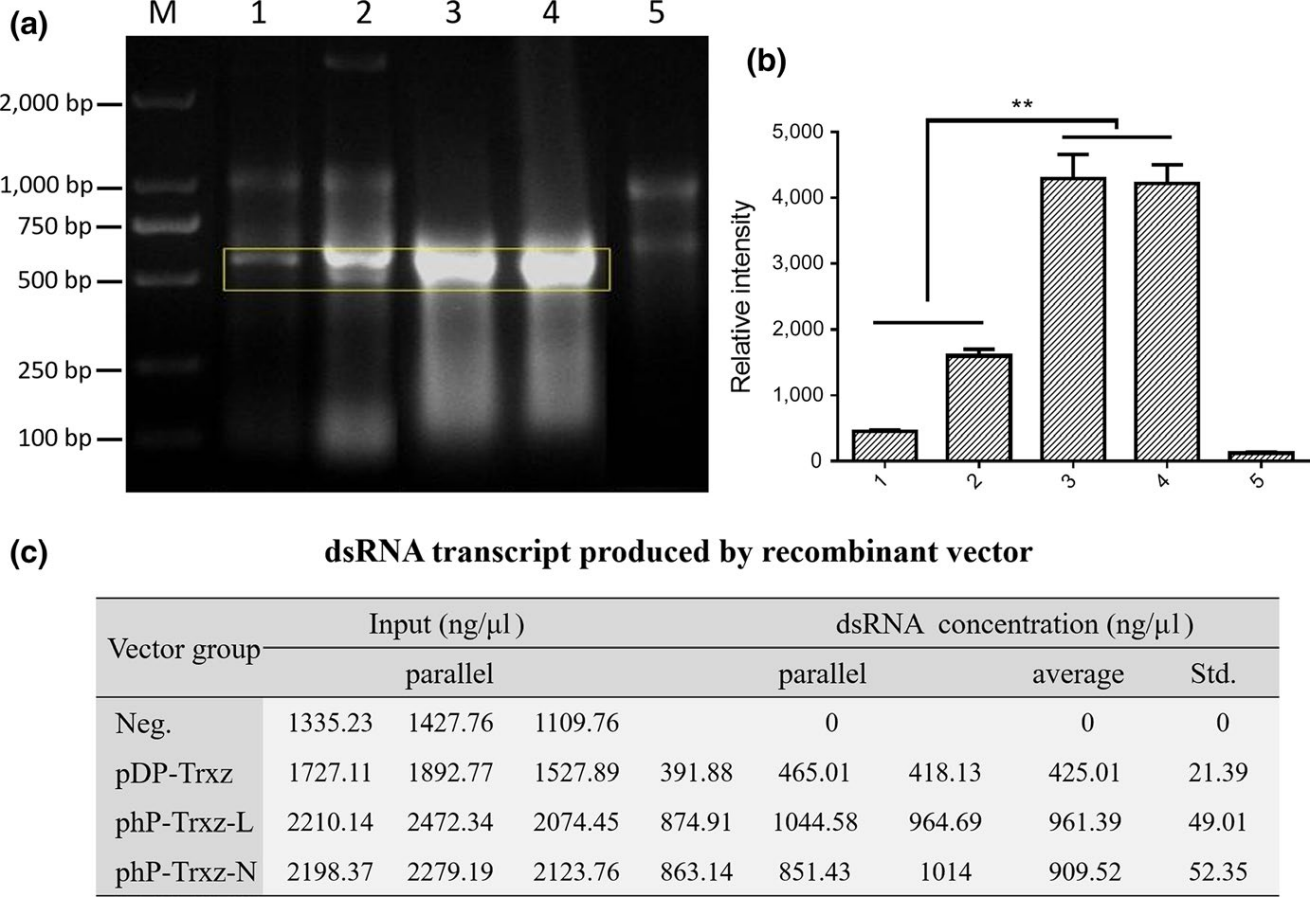
Comparison of recombination vector transcriptional efficiency. (a) Lanes 1 and 2: Transcript dsTrxz produced by pDP‐*Trxz* recombinant vector in the independent repeated trials; Lane 3: Transcript hpTrxz‐L produced phP‐*Trxz*‐L recombinant vector; Lane 4: Transcript hpTrxz‐N produced phP‐Trxz‐N recombinant vector; Lane 5: negative control vector pDP expressed in M‐JM109‐LacY. (b) Quantitative analysis of gel bands demonstrated that dsTrxz transcript produced by phP vector in lanes 3 and 4 is significantly higher than pDP vector in lanes 1 and 2. The bands intensity was evaluated with ImageJ. **p < 0.01 (one‐way ANOVA). (c) The *Escherichia coli* transfected with recombinant plasmid was collected for total RNA extraction. Total nucleotides concentration was analyzed on an Epoch Spectrophotometer system (BioTek) and the dsRNA concentrations were calculated via experimental groups and the control group

To visually compare the differences in the ability of the pDP and phP vectors to express dsRNA, pDP and phP recombinant vectors were transferred into M‐JM109‐LacY for expression. Gel electrophoresis was performed to separate dsRNA segments after the collection of bacteria and crude extracts of total RNA. The result is shown in Figure 2. The dsRNA products appeared around 550 bp. The hpTrxz in the 3rd and 4th lanes was much brighter than the dsTrxz in the 1st and 2nd lanes, and the bands were also wider (Figure 2a,b). This indicates that the dsRNA produced by phP transcription (corresponding to hpRNA) was much higher than the fully‐matched dsRNA produced by pDP (corresponding to dsRNA) during the same period. dsRNA transcript from pDP‐*Trxz* vector in lanes 1 and 2 showed different intensity, suggesting the poor stability of pDP vector compared to the phP vector (Figure 2a,b). In addition, calculated results of dsRNA concentrations correspond to the gel bands density results (Figure 2c). These results suggest that the phP vector performed better and produced more dsRNA under the same conditions than the pDP vector. The transcription efficiency of the phP vectors is much higher than the pDP vector. So phP‐Trxz‐N/L recombinant vectors were chosen to perform subsequent experiments.

### 3.3 Enzymatic purification of dsRNA crude extracts

**Figure 3:**
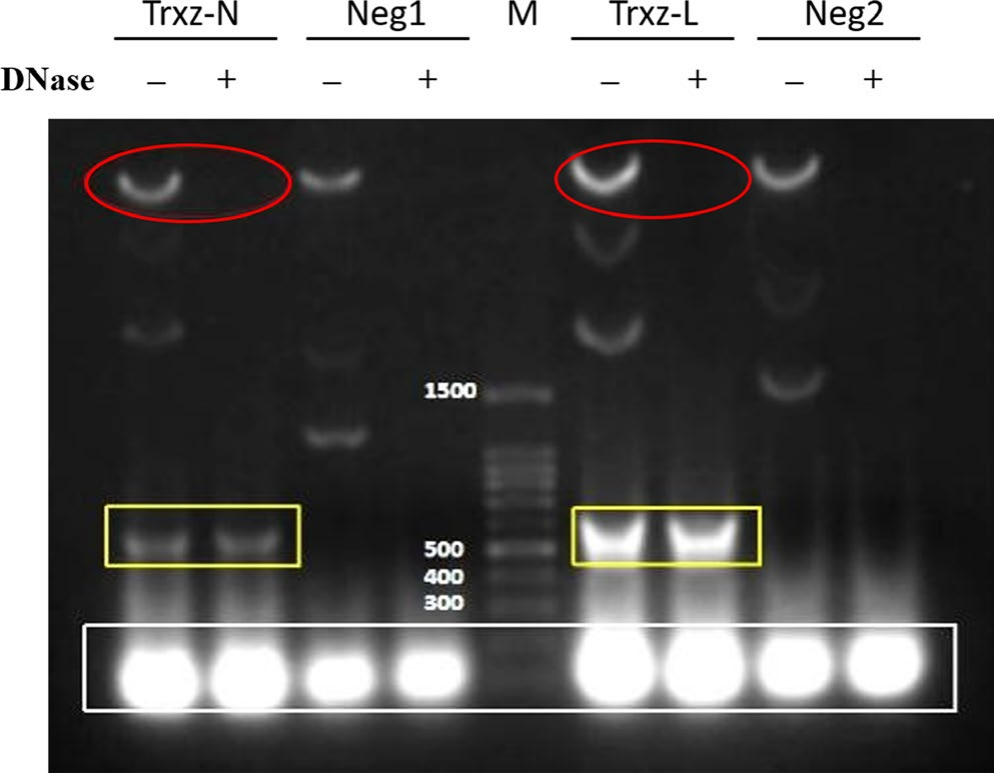
Enzymatic purification of dsRNA crude extracts. Gel electrophoresis evaluated the dsRNA crude extracts from M‐JM109‐LacY, Trxz‐N, and Trxz‐L treated with (+) or without (−) DNase for 15 min. Neg1, extracts with control phP vector in M‐JM109‐LacY. Neg2, extracts of M‐JM109‐LacY

To examine the effect of enzymatic purification of dsRNA, in this study, crude extracts of the dsRNA were dissolved with buffer RDD for enzymatic digestion, with the addition of DNase. The dsRNA was then purified by gel electrophoresis. As shown in Figure 3, the DNase successfully eliminated the plasmid and genome contamination (red circle) in the dsRNA sample compared with the control, and the target dsRNA product with a size of around 550 bp was effectively purified (yellow box). A large number of white bright bands were present in the white box with a size of around 100 bp, which may be the intracellular ribosomal RNA. The results showed that highly purified dsRNA can be obtained through the use of DNase enzymatic hydrolysis of crude extracts.

### 3.4 Immersion of roots in hpTrxz‐N inhibits *A. thaliana* development

**Figure 4:**
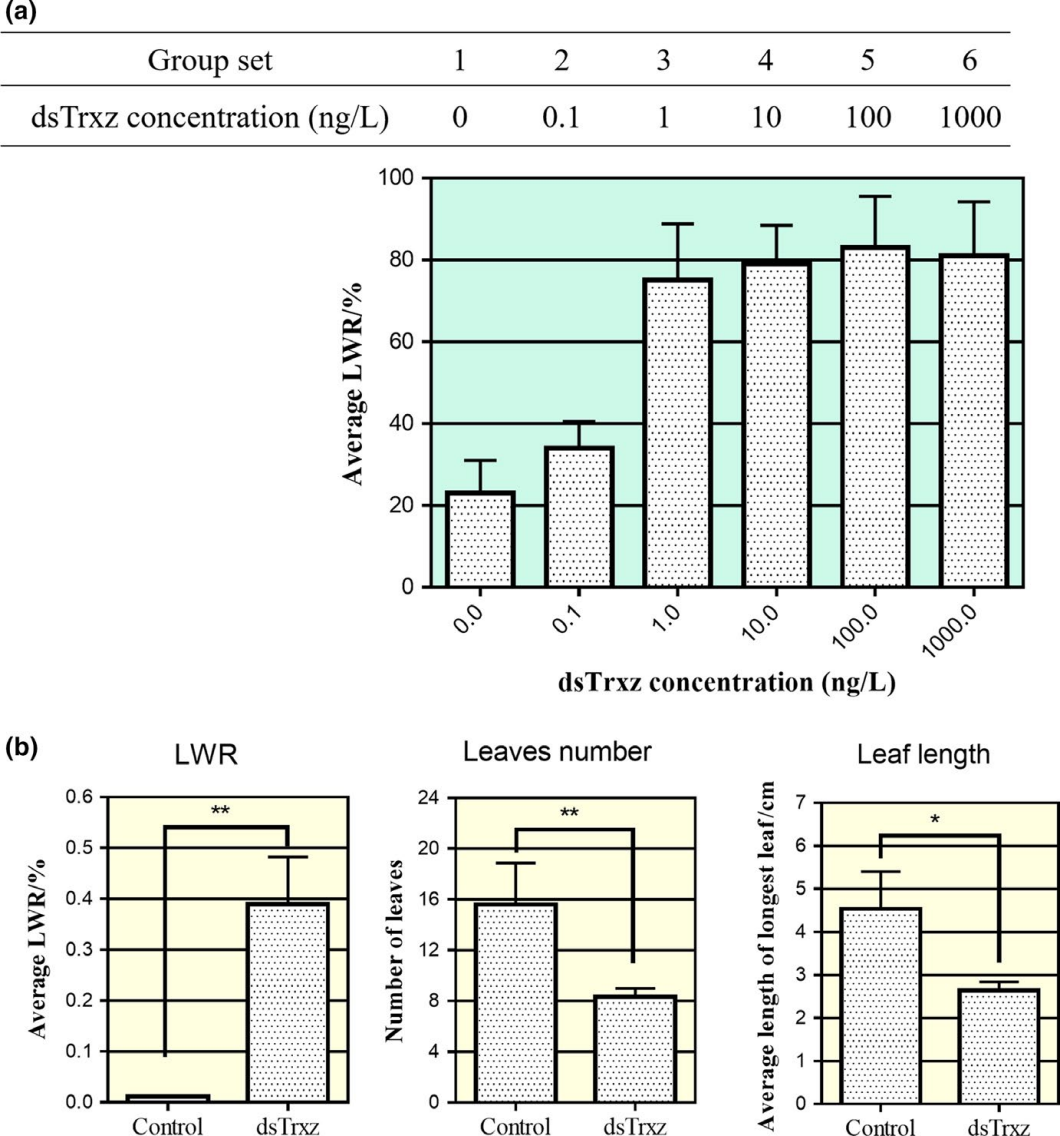
Immersion of roots in hpTrxz‐N inhibits Arabidopsis development. (a) The same batch of *Arabidopsis* seedlings grown on MS medium for 7 days after germination was divided into six subgroups. Each subgroup was treated with different concentrations of dsTrxz (hpTrxz‐N) with a concentration gradient of 0, 0.1, 1, 10, 100, and 1,000 ng/L. The average leaf whitening rate (LWR) was observed after treatment for 7 days. (b) *Arabidopsis* was cultured in liquid medium containing 10 ng/L hpTrxz‐N. After 4 weeks of growth, the difference in morphology between the *Arabidopsis* in the experimental and control groups was measured, including LWR, leaf number, and maximum leaf mean length. *p < 0.05, **p < 0.01 (one‐way ANOVA)

To find the effective concentration for inducing RNAi in *A. thaliana*,* A. thaliana* seedlings grown for 7 days after germination on MS medium from the same batch were divided into six groups. Each group was treated with a gradient concentration of dsTrxz (hpTrxz‐N) at 0, 0.1, 1, 10, 100, and 1,000 ng/L. After treatment for 7 days, the average leaf whitening rate (LWR) was observed. As shown in Figure 4a, the LWR in the experimental group was significantly higher than in the control group, and leaf whitening rate reached a stable value when the dsRNA (hpTrxz‐N) concentration was higher than 1 ng/L. Therefore, 1 ng/L was chosen as the treatment concentration in the subsequent study.

To further observe the dsRNA‐mediated interference during the growth of *A. thaliana*,* A. thaliana* was cultured in a liquid medium supplement with 1 ng/L hpTrxz‐N. The difference in morphology was monitored between the experimental and control groups after 4 weeks, including LWR, leaf number, and maximum leaf mean length. As shown in Figure 4b, root immersion culture with 1 ng/L hpTrxz‐N significantly impacted the growth and development of *A. thaliana*, including inhibiting the development of new leaves, causing weak plant development, leaf whitening, and other symptoms (Figure 4b). The results indicate that hpTrxz‐N could be absorbed through *A. thaliana* roots and continue to trigger *Trxz* gene silencing, so as to inhibit the normal development of *A. thaliana*. These results demonstrated that in vitro expressed dsRNA has the ability to trigger target gene silencing.

### 3.5 Delay of dsRNA degradation by BioClay

The exposed dsRNA molecules are highly susceptible to degradation by the environmental RNase under natural conditions, which restricted the application of the dsRNA direct delivery method (Scott et al., 2013). The emergence of nanomaterial LDH provides an efficient way to extend the life of dsRNA in the natural environment. The LDH nanosheets are inorganic layered material with a negative charge (see Appendix Figure A1) (Gasser, 2009) and have the ability to deliver biomolecules into intact plant cells (Bao, Wan, & Baluska, 2017; Mitter et al., 2017). In the complex that formed through the combination of LDH and dsRNA, LDH released dsRNA molecules continuously and slowly (Mitter et al., 2017).

**Figure 5:**
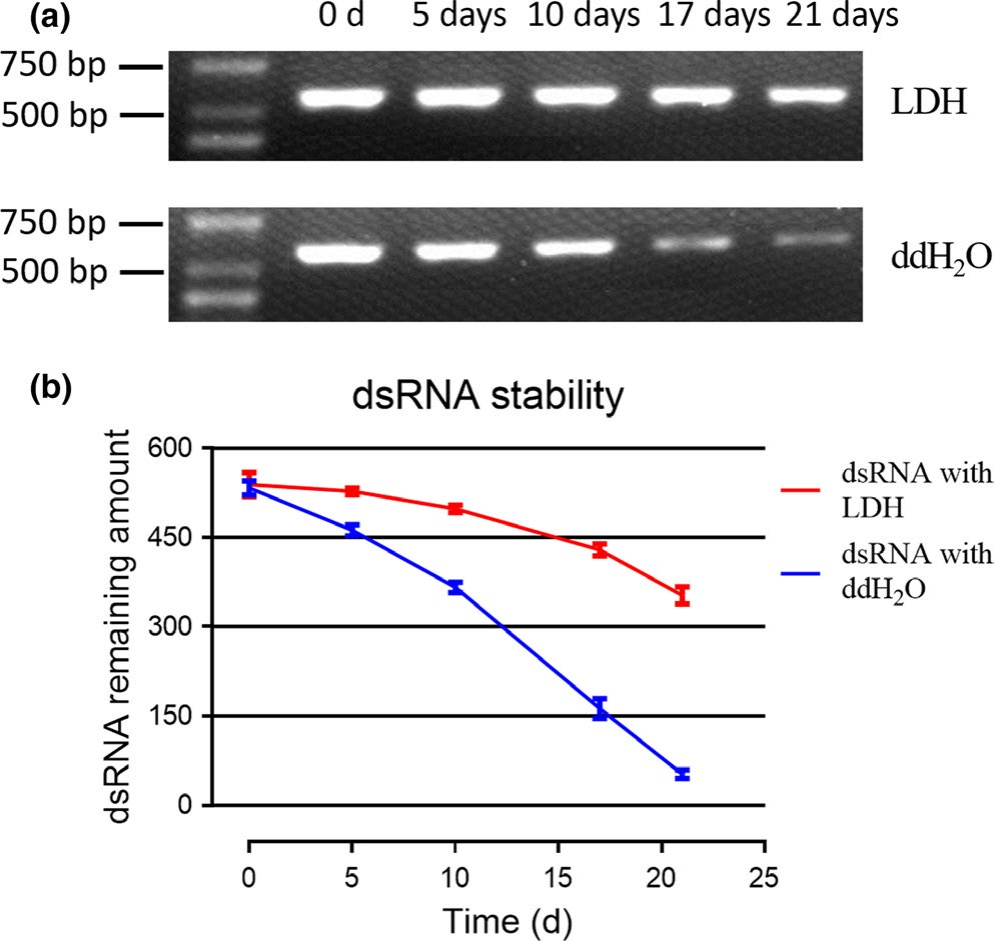
BioClay delays dsRNA degradation. (a) The purified dsTrxz samples were separately dissolved in LDH (with a particle size of 172 nm) and double distilled water (ddH_2_O) to a final concentration of 20 ng/μl. All samples were left to stand at room temperature and collected on days 0, 5, 10, 17, and 21 for agarose gel electrophoresis. (b) Quantitative analysis of dsRNA concentration demonstrated that dsTrxz dissolved in LDH (BioClay) delays dsRNA degradation, more so than in ddH_2_O

In order to verify whether BioClay can delay the degradation of dsRNA, the purified dsTrxz samples were dissolved in LDH solution (with a particle size of 172 nm) and ddH_2_O, respectively, to a final concentration of 20 ng/μl. All samples were kept at room temperature and collected at days 0, 5, 10, 17, and 21 for agarose gel electrophoresis. As shown in Figure 5, the dsRNA showed more rapid degradation in ddH_2_O than in LDH solution after 5 days, suggesting that LDH solution is helpful to protect dsRNA from natural degradation. This method provides a new idea for the storage and transportation of dsRNA.

## 4 DISCUSSION

Nowadays, RNAi technique, mediated by sequence‐specific dsRNA with its function of effectively inducing the suppression or silence of the target genes’ expression, has been widely used in agricultural and bio‐medical fields (Al‐Ayedh et al., 2016; Dutta et al., 2014; Fellmann & Lowe, 2014). Notably, dsRNA‐mediated RNAi in vitro provides an alternative approach for transgenic technology to avoid public panic caused by genetically modified (GM) technology (Li, Guan, Guo, & Miao, 2015). However, dsRNA has a half‐life of about 2 weeks in its natural state and gradually degrades with RNase in the surrounding environment over time. Our study aims to improve dsRNA transcriptional efficiency using a prokaryotic expression system and delay dsRNA degradation using nanomaterial LDH‐mediated BioClay.

This method provides an alternative approach for RNAi in plants transformed with the dsRNA expression vector, through which two types of dsRNA‐producing vectors, pDP and phP, were successfully constructed and dsRNA were both produced by RNaseIII‐deficient *E. coli* M‐JM109‐LacY (Yin et al., 2009) through the use of both recombinant vectors (Figure 1). In addition, our results showed that the single promoter vector phP induces higher efficient dsRNA expression than the pDP vector under the same conditions (Figure 2). This could be because during the transcription process of the pDP vector, the RNA polymerases working in opposite directions may interfere with each other (Garcia‐Muse & Aguilera, 2016; Lin & Pasero, 2017). The number of intracellular RNA polymerases is limited. Compared with phP vectors, the pDP vector requires twice the amount of RNA polymerase to produce a certain quantity of dsRNA. Therefore, the transcription efficiency of the phP series is much higher than that of the pDP vector.

Compared with the traditional annealing method, the main advantage of this dsRNA‐producing method is its accuracy (Lau et al., 2014; Yin et al., 2009), which allows production of large quantities of high‐quality dsRNA at one time. Moreover, studies have confirmed that the higher the concentration of dsRNA, the higher the gene silencing efficiency induced after intake by aphids from their mouthparts (Scott et al., 2013). In order to facilitate the subsequent effect validation of expression products in *A. thaliana*, it is necessary to remove genomes and plasmids from the extracts. Our experimental results show that highly purified and concentrated dsRNA could be harvested by enzymatic hydrolysis assay (Figure 3). This strategy has laid the foundation for studying the direct delivery method of dsRNA/hpRNA based on RNAi.

Previous reports confirmed that citrus trees can directly acquire external dsRNA molecules through root absorption and phloem injection (Hunter et al., 2012). Our root immersion studies demonstrated that in vitro expressed dsRNA using a prokaryotic expression system and enzymatic digestion effectively triggers target gene silencing in *A. thaliana* (Figure 4). Together with previous studies that immersion of *A. thaliana* roots in dsMob1A (the Mob1A gene plays an important role in the organ development and reproduction of plants) significantly inhibited the number and length of roots and the plants blooming (Li et al., 2015), these results indicate that dsRNA irrigation can deliver exogenous dsRNA molecules into plants and regulate plant genomes.

The studies of root immersions in dsRNA also broaden the application of RNAi. Compared with obtaining transgenic plants, the RNAi triggered by dsRNA transcripts in this strategy has obvious advantages. First, this method avoids time‐consuming and costly construction of transgenic plants. Second, direct delivery methods of dsRNA transcripts do not change the target genome, but instead regulates the target gene at the post‐transcriptional level within an effective period of time. This effectively avoids the potential adverse effects of transgenic plants on ecology, such as heterologous encapsidation, complementation, synergy, and horizontal gene transfer among organisms (Tenllado, Llave, & D AZ‐RU Z, J., 2004). We believe that the most obvious advantage of the exogenous delivery method is its controllability. Dependent variables such as shape and duration can be controlled by controlling the amount of dsRNA. Therefore, these methods provide broad application prospects for the future.

## CONFLICT OF INTEREST

The authors declare no conflicts of interest.

## AUTHORS CONTRIBUTION

Z Chen and J He performed the experiments; P Luo and Y Gao analyzed data; X Li and Y Gao designed and supervised the study; Y Gao wrote the paper.

## ETHICS STATEMENT

None required.

## Data Availability

All data are provided in full in the results section of this article.
